# Supplementary material for: Predicting visual function by interpreting a neuronal wiring diagram
Source: Nature. 2024 Oct 2;634(8032):113–23. doi: 10.1038/s41586-024-07953-5 (PMC11446822; doi:10.1038/s41586-024-07953-5)
Supplement: Supplementary file 5 — CRF and ERF predictions for individual Dm3 cells. The CRF is predicted for each target cell by mapping the top five monosynaptic pathways from hexel source types to the target Dm3 type. The ERF is predicted using disynaptic pathways that pass through the top ten intermediary types (ranking from Extended Data Fig. 7). As a set of disynaptic pathways passes from multiple hexel source types through each intermediary to the target, for brevity only one representative starting from a single hexel type is shown, the pathway in the set with maximal strength (red line, Extended Data Fig. 7). The centre of the Tm1–Dm3 CRF (Methods) is indicated by the cyan star, to aid comparison of locations across maps. Next to each map are the maximum and sum over all hexels, in units of 0.01%. In the last panel, all ellipse approximations are shown together for comparison, scaled up by ×3 relative to maps. The dashed ellipse approximates the Tm1–Dm3 CRF, and scale bars indicate one lattice constant and p and q axes. Each file name contains the ID of the target cell in v783. [file 41586_2024_7953_MOESM5_ESM.zip › DataS3/Dm3v/720575940603408608.pdf]

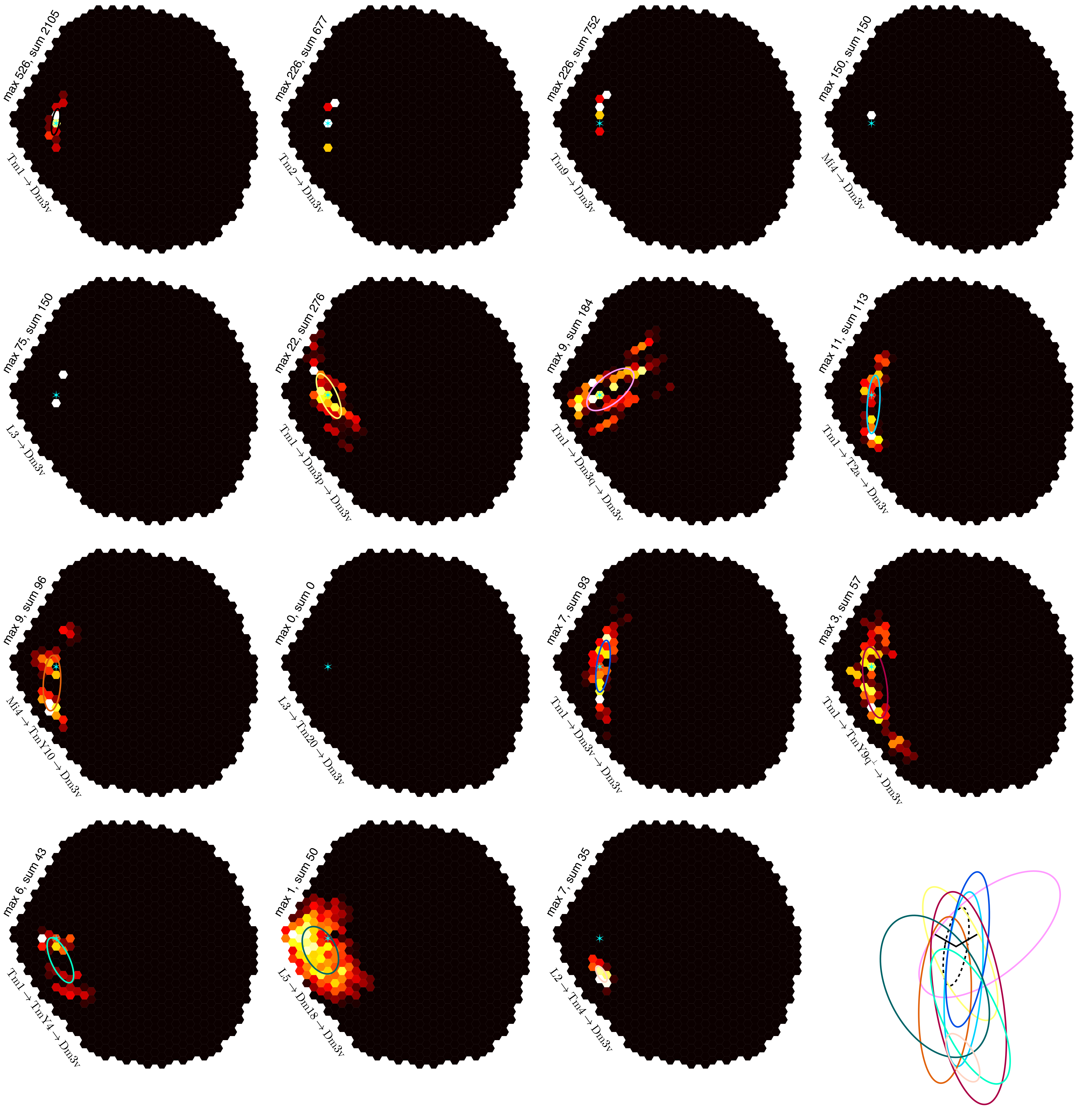
max 526, sum 2105
Tm1 → Dm3v
max 226, sum 677
Tm2 → Dm3v
max 226, sum 752
Tm9 → Dm3v
max 150, sum 150
Mi4 → Dm3v
max 75, sum 150
L3 → Dm3v
max 22, sum 276
Tm1 → Dm3p → Dm3v
max 9, sum 184
Tm1 → Dm3q → Dm3v
max 11, sum 113
Tm1 → T2a → Dm3v
max 9, sum 96
Mi4 → TmY10 → Dm3v
max 0, sum 0
L3 → Tm20 → Dm3v
max 7, sum 93
Tm1 → Dm3v → Dm3v
max 3, sum 57
Tm1 → TmY9q⊥ → Dm3v
max 6, sum 43
Tm1 → TmY4 → Dm3v
max 1, sum 50
L5 → Dm18 → Dm3v
max 7, sum 35
L2 → Tm4 → Dm3v
